# P-57. Staphylococcus aureus Bacteremia in a Tertiary Dominican Hospital: Are We Losing the MRSA Fight?

**DOI:** 10.1093/ofid/ofaf695.286

**Published:** 2026-01-11

**Authors:** Yeison Reyes-Burgos, Ann S Sánchez-Marmolejos, Ricardo Ernesto Hernandez-Landa, Francisco Guzman-Ricardo, Rita A Rojas-Fermín, Anel E Guzmán-Marte, José A Ledesma-Baéz

**Affiliations:** Hospital General Plaza de la Salud, santo domingo, Distrito Nacional, Dominican Republic; Hospital General de la Plaza de la Salud, Distrito Nacional, Distrito Nacional, Dominican Republic; Universidad Ibero Americana, Santo Domingo, Distrito Nacional, Dominican Republic; Hospital General de la Plaza de la Salud, Distrito Nacional, Distrito Nacional, Dominican Republic; Hospital General De La Plaza De La Salud, Distrito Nacional, Distrito Nacional, Dominican Republic; Hospital General De La Plaza De La Salud, Distrito Nacional, Distrito Nacional, Dominican Republic; Hospital General De La Plaza De La Salud, Distrito Nacional, Distrito Nacional, Dominican Republic

## Abstract

**Background:**

*Methicillin-resistant Staphylococcus aureus* (MRSA) bacteremia poses major clinical challenges due to its severity, limited treatment options, and high mortality. Comparative data with methicillin-susceptible *S. aureus* (MSSA) are scarce in low-resource settings. This study aimed to compare their clinical features, management, and outcomes.

**Figure:**
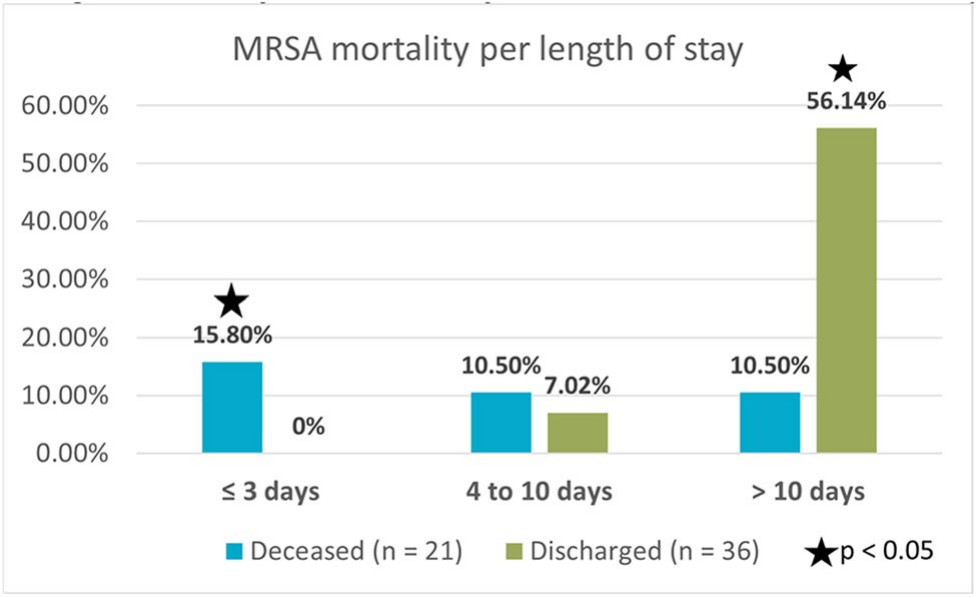

**Figure:**
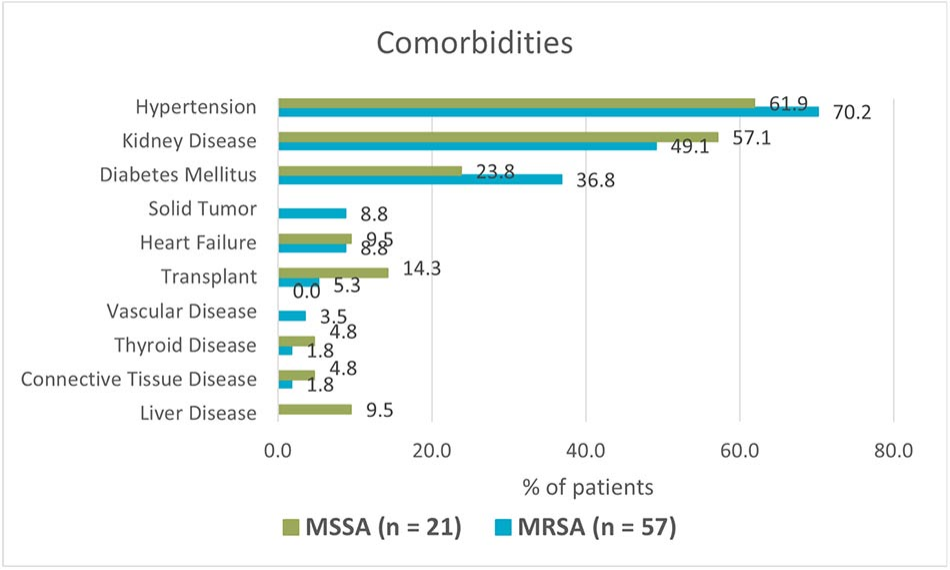

**Methods:**

A retrospective, descriptive and analytical study was conducted on patients with *S. aureus* bacteremia diagnosed between 2023 and 2024 at a tertiary hospital in the Dominican Republic. Clinical, microbiological, and therapeutic data were extracted. Statistical comparisons focused on MRSA using chi-square tests (p< 0.05).

**Figure:**
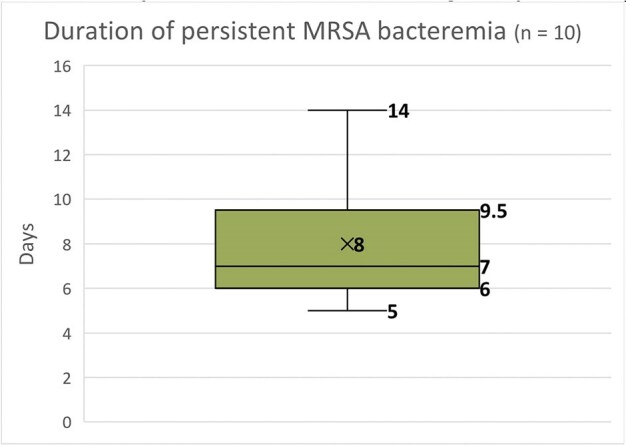

**Figure:**
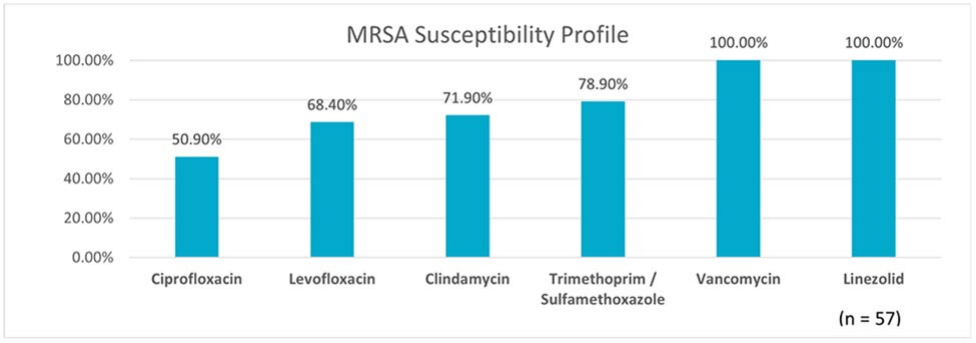

**Results:**

A total of 78 cases of *S. aureus* bacteremia were identified (57 MRSA and 21 MSSA). The median patient age was 54.5 years (IQR 41–66), and 54 (69.2%) were male. The most prevalent comorbidities were hypertension in 70% and chronic kidney disease 49%. Hemodialysis catheters were the most frequent invasive risk factor, present in 23 cases (40%), followed by central venous catheters in 10 cases (18%, p = 0.04). All MRSA isolates were susceptible to linezolid and vancomycin. Susceptibility to Trimethoprim-Sulfamethoxazole was 78.9%, followed by clindamycin (71.9%), levofloxacin (68.4%), and ciprofloxacin (50.9%). Vancomycin therapy was preferred in MRSA cases and oxacillin in MSSA. Persistent bacteremia was found in 10 MRSA cases (17.5%, p = 0.012); among them, four required combination therapy with ceftaroline plus daptomycin. Overall mortality among MRSA patients was 28 (36.8%), with early mortality (within 3 days of admission) occurring in 9 cases (15.7%, p < 0.01).

**Conclusion:**

MRSA bacteremia demands prompt and aggressive management, particularly in patients with invasive devices and significant comorbidities. The high rate of early mortality—within the first 72 hours—despite therapy highlights the severity of disease. Although susceptibility was favorable, persistent cases required combination treatment, reinforcing the need for early clinical vigilance, optimized antimicrobial strategies, and effective source control.

**Disclosures:**

Rita A. Rojas-Fermín, MD, GSK: Honoraria

